# The annual conference of National Cancer Institute - Cairo University "Bridging Gaps in Oncology": InterContinental Citystars Cairo hotel. 3 - 5 November, 2021

**DOI:** 10.1186/s43046-022-00106-7

**Published:** 2022-03-21

## P1 Downregulation of hypoxia-inducible factor 1 α expression inhibits growth and enhances IGF1R inhibitor OSI906 sensitivity in head and neck squamous cell carcinoma cells

### Ashraf Khalil^1,2^, Mark Jameson^1^

#### ^1^Department of Clinical Biochemistry and Molecular Diagnostics, National Liver Institute, Menoufia University, Shebin ELkom, Egypt; ^2^Department of Otolaryngology – Head and Neck Surgery. University of Virginia, Charlottesville, Virginia, USA

##### **Correspondence:** Ashraf Khalil (ashkalil2010@gmail.com)

**Background**

Hypoxia-inducible factor 1 α (HIF‑1α) is a central regulator for cells to adapt to low cellular oxygen levels, is also often overexpressed and activated in many human cancers. HIF‑1α mediates the primary transcriptional response of a wide range of genes in response to hypoxia. Insulin‑like growth factor‑1 receptor (IGF‑1R) is a cell membrane receptor involved in cell proliferation is expressed in the head and squamous cell carcinoma.

**Aim**

To detect the effect of HIF‑1α downregulation on the sensitivity of squamous cell carcinoma (SCC) to small molecule IGF1R inhibitor OSI-906.

**Methods**

A lentivirus-mediated doxycycline-inducible pTRIPZ short hairpin RNA micro (shRNAmir) plasmid targeting HIF‑1α was transfected into two head and neck squamous cell carcinoma (HNSCC) cell lines to silence HIF‑1α expression and to assess the effect of its downregulation on cell proliferation and sensitivity to IGF1R inhibitor. A nude mice animal model was developed by the transfection of a luciferase gene into the inducible shRNA HIF‑1α cells and growing tumors by direct inoculation of the cells in the tongue. Tumor growth was determined by detecting the bioluminescence signal by the advanced IVIS 200 technology (Figure 2d).

**Result**

**Figure 1:**
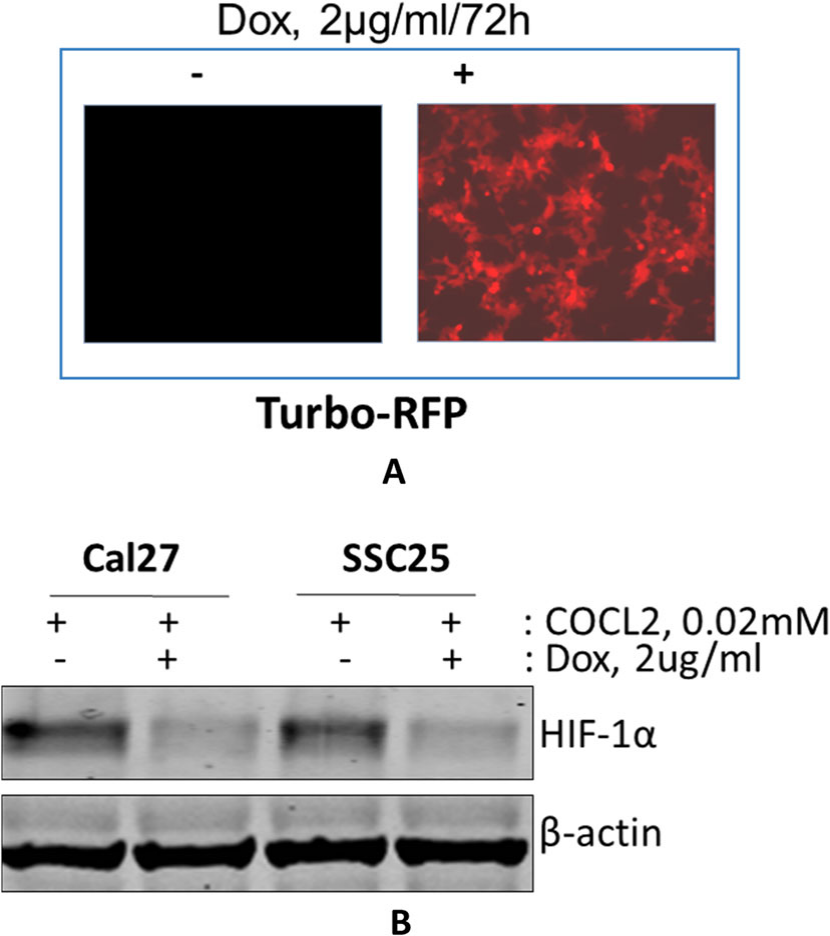
Lentivirus-mediated shRNA efficiently inhibited the expression of HIF‑1α in head and neck squamous carcinoma Cal27 and SCC2 cells. **A**. Fluorescence microscopic picture of the expressed reporter Turbo-RFP protein as a marker of successful transfection and doxycycline indelibility. Lentivirus doxycycline-inducible pTRIPZ-shRNAmir constructs directed against HIF‑1α used to transfect Cal27 and SCC25 HNSCC cells. The construct also contains a doxycycline-inducible TurboRFP as a reporter gene co-expressed with the shRNA sequence. **B.** Immunoblot with anti- HIF‑1α showed that doxycycline-induced HIF-shRNAmir reduced the HIF‑1α protein level by 90% relative to the untreated cells. Cobalt chloride at 2mM was added for 16h to induce hypoxia in the cell and induce HIF‑1α.

**Figure 2:**
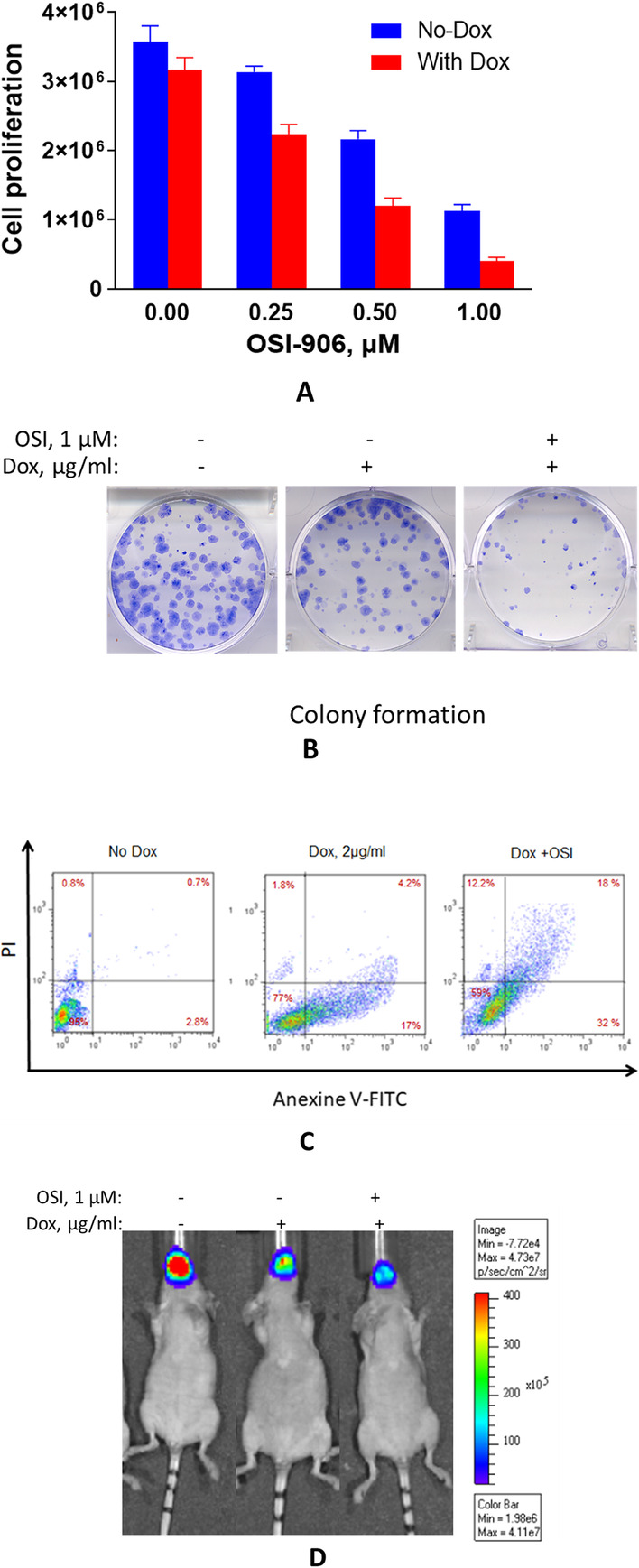
Downregulation of HIF‑1α suppress cell growth, colony formation, increased apoptosis, and the sensitivity of cells to IGF1R inhibition. **A.** Cells transfected with the silencing shRNAmir HIF‑1α were treated with 2 μg/ml doxycycline for three days, then with OSI901 as indicated, and cell proliferation was determined at 72 h using alamarBlue. **B.** Colony formation assay at 14 days. **C.** Apoptosis assay 72h by Annexin V and PI by flow cytometry. **D.** Tumor detection and quantification by Bioluminescence imaging.

Downregulation of the expression of HIF‑1α (Figure1 a-b) led to an increase in the sensitivity of head and neck squamous cell carcinoma to the OSI-906 IGF1R small molecule inhibitor (Figure 2a-b) by a mechanism involving the increase in apoptosis (Figure 2c).

**Conclusion**

The preliminary findings suggest that co-targeting of IGF‑1R and HIF‑1α may represent a novel approach for resistant head and neck tumors.

This study received supported from the National Institutes of Health (NIH) (K08 grant DE019477) (M.J.J.) and the University of Virginia (UVA) Cancer Center/UVA Department of Otolaryngology–Head and Neck Surgery Pilot Project Grant (M.J.J./D.G.G.). The authors have no other funding, financial relationships, or conflicts of interest to disclose.

## P2 Prognostic implication of MYC/BCL2 expressions in patients with diffuse large B-cell NHL

### Thoraya M Abdel Hamid^1^, Ayman A Gaber^1^, Raafat M Abdelfattah ^1^, Dina A Algamal ^2^, Ghada M Abdelsalam^3^

#### ^1^ Medical oncology Department National Cancer Institute Cairo University, Egypt; ^2^ Medical Oncology Department Tanta Cancer Center, Egypt; ^3^Pathology Department National Cancer Institute Cairo University, Egypt

##### **Correspondence:** Thoraya M Abdel Hamid (thorayaabdelhamid@yahoo.com)

**Objectives**

To study the frequency of C-MYC/BCL-2 in adult Diffuse Large B Cell Lymphoma (DLBCL) patients presented to Egyptian National Cancer Institute and Tanta Cancer Center and to classify these patients into GC & ABC according to CD-10, BCL-6 and MUM-1. Also to assess the correlation between the presence of these prognostic markers and treatment outcome, overall survival and disease free survival.

**Background**

Non-Hodgkin lymphoma is the 7th most commonly diagnosed cancer among men and women; diffuse large B-cell lymphoma represents 32% of non-Hodgkin's lymphoma (NHL) cases. In Egypt, NHL is the 5th most common cancer.

**Methods**

A prospective study that included 100 adult patients with newly diagnosed and pathological confirmed Diffuse Large-B Cell Lymphoma (CD20+) who were treated by R- CHOP regimen presenting to Egyptian National Cancer Institute and Tanta Cancer Center between the period of January 2014 and December 2016 and followed up till January 2019. Detection of CD10, BCL6, MUM1, C-MYC and BCL2 by immunohistochemistry as prognostic markers. The following data were studied: patient's characteristics, treatment outcome and prospective overall survival (OS) and disease free survival (DFS). The current study was approved by NCI Institutional Review board (IRB no. IRV00004025). An informed consent was obtained from each patient.

**Results**

Age ranged from 25-68 years (median age was 49 years). Male cases were 52(52%) while female were 48(48%). Most of our patients presented with advanced stage (42% stage III and 25% stage IV) and (45%) of patients had bulky disease. Extra nodal sites were detected in 42% and patients presented with B-symptoms were 59%. The revised IPI showed that 4% had low IPI, 63% had intermediate IPI and 33% had high IPI. Chemotherapy response showed that 78 patients (78%) had CR response. CR response was lower in the double hit group (45%) while there is no difference between germinal center and non- germinal center groups. Only 21 patients were relapsed. The most chemotherapy toxicity was neutropenia in 29 patients (60.41%) and vomiting in 19 patients (59.37%), most of them were grade I and II. The median disease-free survival (DFS) was 49 months while 2-year and 4-year DFS was 89.2 %, and 59.9 respectively. The median overall survival (OS) was 51.8 months while the 2-year and 4-year OS was 86% and 85% respectively. The germinal center was 35 cases (35%) and activated B-Cell was 65 cases (65%). Patients with double hit were 20 cases (20%), only 5 patients of them were germinal center while 15 patients were activated B-cell. There were better DFS in germinal center group and in non-double hit group. Within the double hit patients there were no statistically significant differences in overall survival between both groups (germinal and non- germinal). There were better overall survival in non- double hit group. Independent factors affecting response to chemotherapy were bone marrow involvement, extranodal involvement, IPI and bulky disease while those affecting disease free survival were germinal center and non-double hit groups and independent factors affecting overall survival were extranodal involvement and IPI.

**Conclusion**

Patients with double hit lymphoma had poor overall survival and disease free survival. There is no difference in overall survival between germinal center and non- germinal center group while the disease free survival was better in germinal center group. The CR response was lower in double hit group while there is no difference between germinal center and non- germinal center group.

**Keywords**

Diffuse large B-cell lymphoma, double hit lymphoma, germinal center and Activated B-cell

**Ethical committee approval:** The current study was approved by National Cancer Institute Institutional Review board (IRB no. IRV00004025).

**Consent to participate:** An informed consent was obtained from each patient.

## P3 Clinical outcomes of pediatric-inspired chemotherapy protocol in adolescent and young adults (AYAs) Acute Lymphoblastic Leukemia patients

### Thoraya M Abdel Hamid^1^, Mossad M El Gammal ^1^, Toqa H Mansour ^1^, Mohamed A Samra ^1^, Enas M Radwan^2^

#### ^1^Medical Oncology Department, National Cancer Institute - Cairo University, Cairo, Egypt; ^2^Clinical pathology Department, National Cancer Institute – Cairo University, Cairo, Egypt

##### **Correspondence:** Thoraya M Abdel Hamid (thorayaabdelhamid@yahoo.com)

**Background**

The overall survival for adolescents and young adults (AYA) with Acute Lymphoblastic Leukemia (ALL) continues to lag behind that of younger children. Many retrospective and prospective studies reported better outcomes with the pediatric-inspired protocols when compared to the adult protocol.

**Aim**

This retrospective study aims to evaluate the efficacy and safety of adopting the pediatric-inspired ALL chemotherapy protocol in treating AYA patients diagnosed with ALL and to compare the clinical outcome with other adult ALL chemotherapy Protocol.

**Results**

Through this period, 169 patients were assessed and treated. The median age was 26years (range 18-39 years) with male to female ratio of 1.86:1. Most of the patients (n=108) had received the modified Dana Faber Protocol. Seven patients had received the Total XV protocol, while 54 cases had received modified GMALL protocol. B-cell ALL was reported in 120 patients (72.7%), of which(C-ALL: 18.8%, Pre B: 50.3%, Pro B: 3.5%), while 45 patients (27.3%) diagnosed with T-cell ALL (Early T: 14.5%, Intermediate T: 10.9% and Late T: 1.8%). Molecular and cytogenetic analysis for patients with B-ALL showed t(9;22) in 26.7%, t (4;11) in 4.2% while t (12;21) in one case only(0.8%). The pediatric-inspired regimens were well tolerated among our patients. When compared to the adult- protocol, the pediatric-inspired regimens were associated with significantly higher DFS (33 vs 18.8 months, p=0.046), While the OS was (26 vs 21 months, p=0.176), and CR rates (91% vs 86%, p=0.515). Also, the pediatric-inspired regimens were associated with lower incidence of septic shock during induction (24% vs 39%, p=0.013), lower incidence of early mortality (20% vs 33%, p=0.059), and shorter period of hospital stay during induction (Mean 31 vs 40 days, p<0.001).

**Conclusion**

- The pediatric-inspired chemotherapy regimens were well tolerated for AYA patients, and it was associated with better outcomes.
- AYA patients should be treated as a unique category of ALL patients with major concerns regarding treatment-related long-term adverse events.
- Continuous sincere efforts should be paid towards improving patients’ adherence to treatment and management of treatment-related adverse events.

**Keywords**

Leukemia, Lymphoblastic, Adolescents and Young Adult

**Ethical committee approval:** The study was approved by the Institutional Review Board (IRB) NCI- Cairo University.

**Consent to participate:** it is a retrospective study with no intervention needed, no special informed consent but the study approval consent was taken by NCI Cairo University.

## P4 Effect of nutritional status on survival of Egyptian patients with gastrointestinal malignancies

### Dalia A Mohammed^1^, Iman A Abdelgawad^2^, Mohamed Hassany^3^, Manar M Moneer^1^, Inas A Elattar^1^, Rasha M Allam^1^

#### ^1^Department of Cancer Epidemiology and Biostatistics, National Cancer Institute, Cairo University, Egypt; ^2^Department of Clinical Pathology and Oncology Laboratory Medicine, National Cancer Institute, Cairo University, Egypt; ^**3**^National Hepatology and Tropical Medicine Research Institute, General Organization For Teaching Hospitals and Institutes, Ministry Of Health And Population, Egypt

##### **Correspondence:** Manar M Moneer (manar_moneer@yahoo.com)

**Background**

Malnutrition is a serious but underdiagnosed problem in cancer patients. Malnutrition may negatively impact treatment outcome, quality of life, and performance status of these patients. This study aimed to evaluate the impact of nutritional status of hospitalized patients with stomach and colorectal malignancies on survival.

**Methods**

This cohort study included 213 patients (93 stomach and 120 colorectal cancers) enrolled during the period from February 2018 to October 2019. The study was approved by the Institutional Review Board at NCI, CU (approval No: 201617063.3), and all participants signed informed consent. Nutritional status was assessed using Mini Nutritional Assessment (MNA) questionnaire. Overall survival (OS) and progression-free survival (PFS) were calculated at one year.

**Results**

According to MNA score: 43.7% of all patients were malnourished; 49.5% of stomach and 39.2% of colorectal cancer patients. Colorectal cancer cases had better survival rates than stomach cancer cases (*p*<0.001). The independent factors that significantly affect OS were the cancer type and nutritional status. Tumor type was the only independent factor affecting PFS.

**Conclusion**

Malnutrition is higher in stomach cancer cases than in colorectal cancer cases, but the difference was not statistically significant. Colorectal cancer cases had better overall survival rates than stomach cancer cases. Malnourished had a worse survival than well-nourished.

**Keywords**

Malnutrition; Mini Nutritional Assessment; Gastro-Intestinal Cancer, Survival

**Ethical committee approval:** The study was approved by the Institutional Review Board at NCI, CU (approval No: 201617063.3).

**Consent to participate:** All participants signed the informed consent.

## P5 Characterization of COVID-19 disease in cancer patients, single institute experience, low income setting

### Ahmed K Bayoumi^1^, Lobna Refaat^2^, Eman Z Kandeel^2^, Mona S Abdellateif^3^, Medhat M khafagy ^4^

#### ^1^Assistant lecturer of Pediatric Oncology and Hematology, National Cancer Institute, Cairo University, Cairo, Egypt; ^2^Professor of Clinical Pathology, National Cancer Institute, Cairo University, Cairo, Egypt; ^3^Lecturer of Medical Biochemistry and Molecular Biology, Cancer Biology Department, National Cancer Institute, Cairo University, Cairo, Egypt; ^4^Professor of Surgical Oncology, National Cancer Institute, Cairo University, Cairo, Egypt

##### **Correspondence:** Ahmed K Bayoumi (ahmed.kamal@y7mail.com)

**Background**

The infectivity of COVID-19 and the variation of clinical behavior including fatal cytokine storms are considered one of the challenges nowadays. Recent reports showed that patients with an active or ongoing treatment for cancer are at a high risk of infection and COVID-19-related complications.

**Methods**

49 patients admitted to NCI with different tumor types infected with COVID-19 were investigated as regards the clinical, pathological and laboratory characteristics, associated risk factors, as well as the course of hospital admissions (Tables 1-2 ).

**Results**

Patients with hematological malignancies represented 30/49 (61.2%) (Table-1). The current study showed that 16 (32.7%) had multisystem organ failure and septic shock. Mortality was encountered in 14 patients (28.6%) within 15 (IQR: 2-34) days of covid-19 infection. Patients who had median serum ferritin of 2950 (range1000- 10000) ng/L, showed increased incidence of mechanical ventilation and death. Similarly, patients with increased D dimer to a level of 1.2 (.9-1.3) ng/ml, had a significantly increased incidence of death. Patients with progressive or active cancer stages showed almost the same rate of COVID-19 infection in comparison to patients with stationary, remission or regressive disease. ICU admission was significantly increased in cancer patients infected with COVID-19 after surgery or who were on chemotherapy (Table-3).

**Conclusions**

We can conclude that cancer patients infected with COVID-19 are at increased risk of adverse events and mortality, especially those receiving chemotherapy or underwent surgical procedure. Therefore, COVID-19 cancer patients should attain special care regarding the anticancer treatment and the other supportive measures to augment their immune system. Initiation of proper cancer treatment modality in newly infected patients with COVID-19 should mainly depend on the severity of viral infection, clinical condition, and finally oncological emergencies. Deaths in cancer patients with concomitant COVID-19 infections was mainly attributed to associated intensified treatment as well as gram negative sepsis.

**Keyword**

Cancer, mortality, COVID-19

**Consent**: Informed consent to participate in this study was obtained from participants (or their parent or legal guardian in the case of children under 16).

**IRB – ethical committee approval:** Project funded by Cairo University number :10/2020 – Ethical committee approval at the National Cancer Institute, Cairo University: May/2020.

**Conflicts of interest:** I have no potential conflict of interest to report

**Table 1:** Clinical features of the assessed COVID-19 infected cancer patients

Variables		Frequency	Percent (%)
**Patients**	Paediatric	15	30.6
	Adult	34	69.4
**Age**	Paediatric (mean±SEM)	7.6±1.39	
	Median (range)	7.5 (1-17)	
	Adult (mean±SEM)	51.3±2	
	Median (range)	52 (23-67)	
**Gender**	Male	27	55.1
	Female	22	44.9
**Comorbidities**	Diabetes mellitus	11	22.4
	Cardiac disease	12	24.5
	Renal disease	12	24.5
**Type of malignancy**	AML	13	26.5
	ALL	9	18.4
	Lymphoma	8	16.3
	HCC	1	2.0
	Synovial Sarcoma	1	2.0
	Breast cancer	3	6.1
	CRC	3	6.1
	Squamous cell carcinoma	1	2.0
	Multiple myeloma	1	2.0
	Cholangiocarcinoma	1	2.0
	Neuroblastoma	1	2.0
	Thyroid	1	2.0
	Urinary bladder cancer	3	6.1
	Ewing sarcoma	1	2.0
	Renal cell carcinoma	1	2.0
	Wilms tumour	1	2.0
**Disease state**	Disease progression	26	53.1
	Remission or responding	19	38.8
	Stationary	4	8.1
**Clinical FEVER**	No	6	12.2
	Yes	43	87.8
**GIT**	No	19	38.8
	Yes	30	61.2
**Chest symptoms**	No	2	4.1
	Yes	41	83.7
	NA	6	12.2
**Bony Pains**	No	39	79.6
	Yes	10	20.4
**Loss of weight**	No	42	85.7
	Yes	7	14.3
**Loss of smell**	No	44	89.8
	Yes	5	10.2
**Sore throat**	No	47	95.9
	Yes	2	4.1
**Dialysis**	Yes	2	4.1
	NA	47	95.9

**Table 2:** Severity of studied patients and chest findings

Variables		Frequency	Percent (%)
**COPD**	No	48	98.0
	Yes	1	2.0
**Possible fungal chest infection**	No	31	63.3
	Yes	18	36.7
**CT Chest**	GGO	21	42.9
	GGO, consolidation	6	12.2
	Consolidation	7	14.3
	GGO, nodules	6	12.2
	GGO, consolidation, nodules	3	6.1
	GGO, nodules, cavitation	6	12.2
**Degree of chest affection**	Mild	4	8.2
	Moderate	32	65.3
	Severe	13	26.5
**Follow up CT Chest**	NA	18	36.7
	Regressive (healing)	15	30.6
	Stationary	12	24.5
	ARDS	2	4.1
	Progressive	2	4.1

COPD: chronic obstructive pulmonary disease, GGO: ground glass opacity, NA: not available

**Table 3:** Correlation with the outcome

laboratory and imaging investigations		Mortality n (%)	Ventilator n (%)	ICU admission, n (%)	inotropic support, n (%)
Possible fungal chest infection	no	8 (57.1)	7 (58.3)	17 (63.0)	9 (56.3)
	yes	6 (42.9)	5 (41.7)	10 (37.0)	7 (43.8)
CT chest	GGO	5 (35.7)	4 (33.3)	9 (33.3)	4 (25.0)
	GGO, consolidation	2 (14.3)	2 (16.7)	4 (14.8)	3 (18.8)
	consolidation	1 (7.1)	1 (8.3)	4 (14.8)	3 (18.8)
	GGO, nodules	2 (14.3)	2 (16.7)	3 (11.1)	2 (12.5)
	GGO, consolidation, nodules	1 (7.1)	0 (0.0)	2 (7.4)	1 (6.3)
	GGO, nodules, cavitation	3 (21.4)	3 (25.0)	5 (18.5)	3 (18.8)
Degree of chest affection	Mild (4)	0 (0.0)	0 (0.0)	3 (11.1)	1 (6.3)
	Moderate (32)	3 (21.4)	2 (16.7)	14 (51.9)	6 (37.5)
	Sever (13)	11 (78.6)**	10(83.3)**	10 (37.0)	9(56.3)**
Follow-up CT chest	Regressive (15)	**1 (7.1)**	**0(0.0)**	6 (22.2)	2 (12.5)
	Stationary (12)	**0 (0.0)**	**0(0.0)**	5 (18.5)	2 (12.5)
	ARDS (2)	1 (7.1)	1(8.3)	1 (3.7)	1 (6.3)
	Progressive (2)	2 (14.3)**	2(16.7)**	2 (7.4)	2 (12.5)*
	NA (17)	10 (71.4)	9 (75)	13 (48.1)	9 (56.3)
Second COVID PCR	Negative (7)	1 (7.1)	1 (8.3)	4 (14.8)	2 (12.5)
	Positive (30)	6 (42.9)*	5 (41.7)*	16 (59.3)	9 (56.3)
	NA (12)	7 (50.0)	6 (50.0)	7 (25.9)	5 (31.3)
Liver functions (billirubin)	Normal (28)	3 (21.4)	3 (25.0)	14 (51.9)	5 (31.3)
	High (21)	11 (78.6)**	9 (75.0)*	13 (48.1)	11(68.8)*
Cultures	Blood (25)	6 (42.9)	5 (41.7)	9 (33.3)	8 (50.0)
	Wound (6)	2 (14.3)	2 (16.7)	6 (22.2)**	2 (12.5%)
	No (18)	6 (42.9)	5 (41.7)	12 (44.4)	6 (37.5)
CRP>100	No (7)	2 (16.7)	2 (18.2)	3 (11.5)	1 (6.7)
	Yes (40)	10 (83.3)	9 (81.8)	23 (88.5)	14 (93.3)
Second CRP	No (10)	0 (0.0)	0 (0.0)	6 (25.0)	1 (7.7)
	Yes (31)	11 (100)*	10 (100)	18 (75.0)	12 (92.3)
lymphopenia	<400	8 (57.1)	7 (58.3)	12 (44.4)	9 (56.3)
	>400	6 (42.9)	5 (41.7)	15 (55.6)	7 (43.8)
HB (g/dl)		9 (6-11)	8.5 (6-10)**	10 (6-13)	9 (6-13)
TLC (10^3^/Cmm)		6.5(.2-4000)	6.5(.2-4000)	7(.2-4000)	4(.2-4000)
Neutrophil count		1250(0-21000)	1250(0-21000)	600(0-21000)	1250(0-20000)
Lymphocyte count (at diagnosis)		350(39-2200)	350(39-2200)	700(39-3200)	350(39-3000)
Lymphocyte count (follow up)		455(90-1800)	420(90-1800)	1000(0-3000)	490(90-3200)
Platelets (10^3^/Cmm)		84.5(19-500)	84.5(19-500)	100(18-900)	89.5(19-900)
Ferritin (ng/L)		2900(1000-10000)*	2950(1000-10000)*	2000(900-10000)	2200(1000-10000)
LDH (U/L)		900(0-3000)	900(0-3000)	895(0-3000)	890(0-3000)
D dimer (ng/ml)		1.2(.9-1.3)*	1.2(.9-1.3)	1 (.9-1.3)	1.15(.9-1.3)
ALT (U/L)		200(10-840)	123(10-840)	70(10-840)	125(20-840)
AST (U/L)		111.5 (19-1000)	95(19-350)	90(14-500)	111.5(19-500)
Creatinine (mg/dl)		1.4(.6-2.9)**	1.35(.6-2.9)*	1.3(0-3.5)**	1.35(0-2.9)*

## P6 Malignant obstructive jaundice; review of 232 patients

### Ashraf SZ Abdelhamid^1^, Mohammed G Ramadan^1^, Ali H Mebed^1^, Nelly H Ali Eldin^2^

#### ^1^ Surgical oncology department at National Cancer Institute - Cairo University, Egypt; ^2^ Biostatistics and Cancer epidemiology department at National Cancer Institute - Cairo University, Egypt

##### **Correspondence:** Ashraf SZ Abdelhamid (ashrafsobhy27@yahoo.com)

**Background**

Obstructive jaundice is a common problem in the medical and surgical gastroenterological practice. Malignant obstructive jaundice can be caused by cancer head of pancreas, periampullary carcinoma, carcinoma of the gall bladder and cholangiocarcinomas.

**Objective**

To review the etiological spectrum of malignant obstructive jaundice in NCI Cairo university during a period of 3 years.

**Patients and methods**

Retrospective study included 232 patients who presented with malignant obstructive jaundice. Data were collected from the biostatistics and cancer epidemiology department.

**Results**

Out of 232 patients; 156 (67.2%) were male and 76 (32.8%) were female; the median age of the study population was 49 years (range 19_80years). The commonest cause of malignant obstructive jaundice was pancreatic head cancer, 72% (167/232), followed by the ampullary carcinoma 15% (36/232).The last cause was cholangiocarcinoma12.5% (29/233). Whipple operation or pancreaticoduodenectomy was done in 37(46.2%), triple bypass was done in 15(18.9%) patients in (irresectable tumors or metastatic cases, choledocojuojonostomy (+/-gastrojuojonostomy) was done in 12(15%) patients (irresectable tumors) and laparoscopic assessment was used in 2(2.5%) patients. There were 43(53.75%) patients underwent abdominal exploration and the tumor was irresectable. The commonest cause of morbidity was biliary leakage 3/37(8.1%) cases post Whipple operation. The commonest cause of mortality was leakage (bile and pancreatic) 2/37(5.4%) cases post Whipple operation.

**Conclusion**

Obstructive jaundice is more common among males and cancer head of pancreas is the commonest malignancy.US, ERCP and CT-Scan are important diagnostic modalities for evaluation of patient with obstructive jaundice with ERCP having the additional advantage of being therapeutic as well.

**Keywords**

Obstructive jaundice, ERCP, Ca Head of pancreas, Ca gall bladder

**Ethical committee approval:** The study was approved from IRB Committee at National Cancer Institute – Cairo University.

**Consent to participate:** No informed consent was done as it was retrospective cohort study.

## P7 Determining resectability in pancreatic tumors; review of 70 cases

### Ashraf S Z Abdelhamid^1^, Mohammed G Ramadan^1^, Hussein Okasha^2^, Ali H Mebed^1^, Reda H Tabashy^3^

#### ^1^Surgical oncology department, National Cancer Institute - Cairo University, Egypt; ^2^Gastroenterology & Hepatology Faculty of Medicine - Cairo University, Egypt; ^3^Radiodiagnosis department, National Cancer Institute - Cairo University, Egypt

##### **Correspondence:** Ashraf S Z Abdelhamid (ashrafsobhy27@yahoo.com)

**Background**

Pancreatic adenocarcinoma is one of the most aggressive tumors of the digestive system, with a prevalence of 10%. Despite treatment options involving chemotherapy and radiotherapy, surgical resection offers the only chance for a cure.

**Objective**

To determine the respectability in pancreatic tumors prospectively for 2 years.

**Patients and methods**

Prospective cohort study including 70 patients who presented with pancreatic tumors underwent many imaging modalities like endoscopic US (EUS), MSCT scan abdomen and MRCP. The study was carried out at Faculty of Medicine Cairo University and National Cancer Institute, Cairo University.

**Results**

Out of 70 patients; median age was 55 years (range 32_73 years). Males were 32 (46%) and females were 38 (54%). There were 20 patients with benign disease and 50 patients with malignant disease. The EUS has the highest accuracy reaching 90.0% in detecting malignant pancreatic tumors followed by MRI & CT pancreatic protocol. EUS has the highest accuracy in determining of resectability criteria and vascular invasion (SMV/PV).

**Conclusion**

Early tumor detection and accurate radiological staging are crucial for identifying patients with potentially resectable disease, and avoiding unnecessary surgery in patients with unresectable disease. EUS can clarify locoregional spread when CT/MR are equivocal. The combination of superior detection, good staging, tissue diagnosis, vascular invasion and potential therapy makes EUS guided FNA a cost-effective modality.

**Keywords**

local treatment; Endosonographic; pancreatic tumors

**Ethical committee approval:** The study was approved from IRB Committee at National Cancer Institute – Cairo University.

**Consent to participate:** Informed consent was done before Endoscopic US for each patient.

## P8 Complete mesocolic excision and central vascular ligation in colon cancer surgery, feasibility and outcome

### Mohamed I Abdelaziz (mohamedibrahim555@hotmail.com)

#### General surgery department, Fayoum University hospitals, Fayoum, Egypt

**Background**

Colon cancer continues to be a major health problem worldwide. Being the third most common type of cancer in men and the second in women. Standard treatment of colon cancer is based on surgical resection. An adequate number of lymph nodes harvested are important for a correct stabilization of the disease; thereby the extension of the colonic resection remains controversial. Complete mesocolic excision (CME) with central vascular ligation (CVL) has recently been found to improve oncological outcomes in patient with colonic cancer. Complete mesocolic excision is based on a correct identification of the dissection plan between the mesofascial plane and the retroperitoneal fascia, central vascular ligation of the vessels to remove vertical lymph nodes and resection of the affected colonic segment.

**Methods**

This is a prospective study done at general surgery department of Fayoum University hospitals from January 2015 to January 2019 including 60 patients with operable colonic cancer operated with adequate surgical margin, complete mesocolic excision and high vascular ligation.

**Results**

The number of dissected lymph node was 27.7 ± 4.2 and this number is more than that dissected in the conventional colectomy mentioned in many studies in literature, more over larger mesocolon area, longer distance from vascular high ligation point to intestinal wall, and longer distance from vascular high ligation point to tumor center were observed.

**Conclusion**

Surgery in colon cancer patients remains the only curative treatment and applying the principles of complete mesocolic excision and central vascular ligation in colon cancer surgery can improve cancer outcomes without increase the incidence of postoperative complications.

**Keywords**

Complete mesocolic excision, central vascular ligation, colon cancer surgery.

**Ethical committee approval**: I got the approval of ethical committee of faculty of medicine, Fayoum University (M408/95 on 14/4/2017).

**Consent to participate:** A fully detailed written consent was taken individually from every patient participated in the study.

## P9 Inhibition of dynamins restricts the survival of vasopressin stimulated and PI3K/Akt/mTOR inhibited breast cancer cells

### Samar S. Alkafas^1^, Thoria A. Diab^1^, Samah A Loutfy^2^, Mohamed HM Hessien^1^

#### ^1^Division of Biochemistry, Department of Chemistry, Faculty of Science, Tanta University, Egypt; ^2^Department of Cancer Biology, National research center, Cairo University, Egypt.

##### **Correspondence:** Mohamed HM Hessien (Mohamed.hessien@fulbrightmail.org; Mohamed.hussien1@Science.tanta.edu.eg)

The internalization of agonist activated G-protein coupled receptor (GPCRs) is a part of receptor trafficking. Also, it involves a list of endogenous proteins, where the endosomed ligand-receptors are distant to recycling or degradation as a mechanism of cellular signal management. Dynamins (Dyns) are large GTPase involved in multiple endocytic pathways, including receptor mediated endocytosis. They perform unique tasks including endosome fission, mitochondrial severing and some signaling events. The impact of Dyns inhibition in vasopressin stimulated or AKT/mTOR inhibited breast cancer cells was not addressed. To explore this, Dyns were selectively inhibited by dynsore in triple negative breast cancer cells (MDA MB-231) in which vasopressin receptor-2 (VR2) was agonist activated with exogenous arginine vasopressin hormone (AVP) or PI3K/Akt/mTOR was inhibited by Wortamnin (Wort). The results depicted the cytotoxic effect of Dyn inhibition, where Dynasore, individually promoted autophagic and apoptotic cell death. Apoptosis was developed in 19%, whereas the autophagy marker LC3II protein was observed in 39%. Dyns inhibition in AVP stimulated cells, PI3K/mTOR inhibited cells or both progressively enhanced the apoptotic and autophagic effects. Similarly, 73% of cells were arrested in G0/G1 phase. The activation of Akt into P-Akt decreased by Dynasore and more repression was observed in cells dually treated with Dynasore, AVP, Wort or both. Also, the drug was able to minimize the invasion of cells as indicated by the transwell assay.

Conclusively the results presented Dynamins as anticancer targets in invasive breast cancer cells.

**Keywords**

Dynamines, PI3K/AKT/mTOR, Dynasore, Wortamnnin, AVP, breast cancer

**Ethical committee approval:** The study was approved by the research ethics committee at Faculty of Medicine Tanta University, approval code: 34251/11/20.

## P10 Early deaths in childhood Burkitt’s lymphoma; a major challenge in developing countries

### Hanafy Hafez^1, 2^, Esraa Maged^1^, Eman Naguib^3^, Youssef Madney^1, 2^, Reham Khedr^1, 2^

#### ^1^pediatric Hematology/Oncology, National Cancer Institute, Cairo University, Cairo, Egypt; ^2^pediatric Hematology/Oncology, Children Cancer Hospital Egypt 57357, Cairo, Egypt; ^3^Surgical Pathology, National Cancer Institute, Cairo University, Cairo, Egypt

##### **Correspondence:** Esraa Maged (esraamaged1991@gmail.com)

**Background**

The outcome of childhood Burkitt’s lymphoma (BL) has improved steadily over the past decades through the use of intensive sequential multi-agent chemotherapy regimens with increased concern about increasing toxicity related mortality.

**Aims**

This study objective was to assess incidence and risk factors of early mortality in BL

**Methods**

A retrospective study included all patients 18 years old or younger diagnosed with BL and treated according to the modified LMB 96 protocol at National Cancer Institute, Cairo University from January 2012 to December 2016.

**Results**

A total of 170 patients were diagnosed with BL, with median age of 5 years old. Abdominal presentation was the most common 1ry site (90.0%). Sixty-two (36.5%) patients were diagnosed as early as less than or equal 2 weeks and 108 (63.5%) patients were diagnosed beyond 2 weeks from onset of symptoms. A total of 82 (48.2%) patients presented by disease related emergencies, 49 patients (59.7%) of them with acute renal impairment due to tumor lysis syndrome (TLS), and 33 (40.2%) had intestinal obstruction and/or complications. Throughout the whole treatment duration, 39 (23%) total deaths were reported and 50% of them (n=19) died before 2nd induction (early mortality). Eleven patient (57.8%) of them died in the first week, 6/11 patients died before completing the 1st cyclophosphamide, vincristine, prednisone (CVP) course. The main causes of early mortalities were sepsis in 58% and acute renal failure due to TLS in 42% of cases. The 5-years overall survival (OS) and event free survival (EFS) were 82.9% and 82.8% respectively for the whole group.

**Summary/Conclusion**

Early deaths and treatment related toxic mortalities remain the major challenge affecting outcome in our patients. Delayed time to diagnosis and late presentation remains a major challenge to improve the outcome.

**Keywords**

Burkitt's lymphoma, Childhood, Mortality, Outcome

**Ethical committee approval:** The study was approved by the Institutional Review Board at National Cancer Institute, Cairo University, IRB approval No: 201819019.4.

**Consent to participate:** There were no informed consents from the patients as the study was retrospective and data were collected from the files and it was not requested by the ethical committee.

## P11 Development of a newly confirmatory immunoassay to evaluate anti-viral activity of chitosan nanoparticles (cnps) against SARS-CoV-2 spike protein

### Merna H Emam^1^, Ahmed I Abdel-Salam^1^, Yassmin Moatasim^3^, Mokhtar R Gomaa^3^, Nasra F Abdel Fattah^2^, Fedaa Ali^1^, Hanaa M Alam El-Din^2^, Ahmed Mostafa^3^, Mohamed A Ali^3^, Samah A Loutfy^1,2^ , Amal Kasry^1^

#### ^1^Nanotechnology Research Center (NTRC), the British University in Egypt, El-Shorouk City, Suez Desert Road, Cairo 11837 -P.O. Box 43, Egypt; ^2^Virology and Immunology Unit, Cancer Biology Department, National Cancer Institute (NCI), Cairo University, Fom El-Khalig 11796, Cairo, Egypt; ^3^Center of Scientific Excellence for Influenza Viruses, National Research Centre (NRC), Giza 12622, Egypt

##### **Correspondence:** Merna H Emam (merna.emam@bue.edu.eg)

**Background**

At the current time, development of an effective antiviral agent against Coronavirus Disease-19 (COVID-19) is of huge concern. It takes months and even years for getting a potential anti-viral agents. In this work, we develop an effective confirmatory immunoassay based on using monoclonal antibody and a secondary labelled antibody, to evaluate the anti-viral activity of any suggested antiviral agent against severe acute respiratory syndrome coronavirus-2 (SARS-CoV-2), avoiding laborious culture technique which is not available in many Labs in Egypt. Chitosan nanoparticles (CNPs) were selected to evaluate our newly immunoassay after evaluating its antiviral effect in both in-silico and in-vitro studies.

**Methods**

Docking of CNPs was carried out against spike protein of SARS-CoV-2 and binding scores were calculated. CNPs were prepared using ionic gelation method. They were characterized using Scanning Transmission Electron Microscopy (STEM), Fourier-transform infrared spectroscopy (FT-IR), and Zeta analyzer. Cytotoxicity was performed on Vero E6 using MTT colorimetric assay. Antiviral activity was evaluated using real-time polymerase chain reaction (PCR) assay. The binding affinity between the Spike protein and CNPs was evaluated and assessed using a monoclonal antibody and a secondary antibody conjugated to Alexa Flour 647 dyes protocol. The viral inhibition was assessed using varying concentrations of CNPs (1, 5, 10, 20, 30, and 50 μg/ml).

**Results**

The strength of binding interactions between spike protein and CNPs ligand complexes gave scores at -6.6 Kcal/mol which is the same for Remdesivir (reference drug). CNPs were prepared at sizes of 35 nm with surface charges of 42 mv. The cytotoxic concentration of CNPs that cause death to 50 % of viable cells (CC50) was determined at 135 μg/ml on Vero E6, cellular uptake confirm localization of nanoparticles inside cells. Antiviral activity of CNPs against SARS-CoV-2 reached at 10 μg/ml as evidenced by undetected viral copies/ml using quantitative real-time PCR assay.

**Conclusion**

These results demonstrate that CNPs represent a promising antiviral candidate against entry of SARS-CoV-2 and successfully preventing infection.

**Keywords**

SARS-CoV-2, Spike protein, Binding affinity, chitosan nanoparticles.

- Funding Agency: ASRT- project ID 6901

- There are no clinical samples involved in this research, so the research did not require an approval from ethics committee.

## P12 Predictors and outcome of infection related mortality in pediatric acute myeloid leukemia, febrile neutropenic episodes analysis, single institute experience

### Ahmed Kamal Bayoumi^1^, Reham AbdelAziz Khedr^2^, Samah Mohamed Radwan ^4^ , Alaa Elhaddad^3^

#### ^1^Assistant Lecturer of pediatric hematology and oncology – National cancer institute , Cairo University , Cairo Egypt; ^2^Associate professor of pediatric hematology and oncology, National cancer institute , Cairo University , Cairo Egypt; ^3^Professor of pediatric hematology and oncology - National cancer institute , Cairo University , Cairo Egypt; ^4^ Lecturer of clinical pathology , National cancer institute , Cairo University , Cairo Egypt

##### **Correspondence:** Ahmed Kamal Bayoumi (ahmed.kamal@y7mail.com)

**Background**

Children with acute myeloid leukemia (AML) are at a particularly high risk for infectious complications related to the highly intensive chemotherapy. The aim of the study is to: assess the risk factors, infectious complications and assess outcome of febrile episodes in children with AML at the Pediatric Oncology Department, National Cancer Institute, Cairo University from January 2016 to December 2018.

**Methods**

Infectious complications were evaluated retrospectively in 621 febrile episodes in101 Patients: were divided into survivors and non-survivors according to outcome at end of each episode. Each febrtile episode was interpreted in correlation with infectious complications.

**Results**

Mortality from gram negative bacteremia was 29.9%, in febrile episodes with multidrug resistant gram negative bacteremia: Mortality was 39.2 % .In febrile episodes with multidrug resistant gram negative bacteremia and septic shock. Mortality was 71.8 % (p value <0.001). Mortality was high in early chemotherapy phase (intensive timing). Infection related mortality was 39%. In our institute there is epidemiological shift towards gram negative organisms. In clinically documented febrile episodes: Mortality was 13.2 % (p value <0.001). Mortality rate was 15.3% for patients who presented with pneumonia, as compared to 6 % who hadn’t. (P value = 0.001). It was 25.7% for patients who had typhilitis/colitis, as compared to 9% who hadn’t. (P value <0.001; statistically significant). However, it was 11.7% for patients who had soft tissue infection, as compared to 9% who hadn’t. In clinically documented febrile episodes with multidrug resistant gram-negative bacteremia: Mortality was 42.9% (p value =0.122). Mortality from febrile episodes with ICU admission was 58.5 %. (P value <0.001). (Tables 1-2).

**Conclusions**

Sepsis and septic shock are major causes of mortality. Improved management of sepsis during neutropenia may reduce the mortality of pediatric Acute myeloid leukemia. It is Important to trace the predictors that may impact the outcome of febrile episode.

**Keywords**

Acute Myeloid Leukemia, Gram negative - Predictors, Mortality.

**Conflicts of interest:** I have no potential conflict of interest to report

**Consent:** Informed **consent to participate** in this study was obtained from participants (or their parent or legal guardian in the case of children under 16)

**IRB- ethical committee approval**: From the National cancer institute, Cairo University, 5/2019.

**Table 1:** Summary of Laboratory predictors of mortality

Risk factor	Mortality	p value
CRP more than or equals 90 mg/l	24.4%	*
ANC LESS THAN 500	29.9%	**0.003**
Hgb less than or equals to 7 g/dl	19.9%	0.633
Platelets less than 20000/cc	29.9%	0.299
Liver impairment (grades 3 and 4)	42.2%	0.025
Electrolyte imbalance (grades 3 and 4)	24.1%	**0.003**
Renal impairment	56.8%	0.025
Coagulopathy	41%	**<0.001**

**Table 2:** Summary of Risk factors of Mortality in the studied episodes

Risk Factor	Mortality	p value
Septic shock	**55**%	**<0.001**
Septic shock with MDRO	**72**%	
Cardiac impairment/inotropic support	**65.60**%	
Presence Of Central venous line	18.30%	
Episode duration >18 days	20.50%	
Start of antimicrobial in relation to start of chemotherapy < 16 days	33%	

